# The effect of water restriction on physiological and blood parameters in lactating dairy cows reared under Mediterranean climate

**DOI:** 10.5713/ajas.18.0098

**Published:** 2018-05-31

**Authors:** Amel Benatallah, Faissal Ghozlane, Michel Marie

**Affiliations:** 1 Higher National Veterinary School of Algiers (ENSV), 16000 Issad Abbes, Oued Smar Algiers, Algeria; 2 Department of Animal Science, Higher National Institute of Agronomy (ENSA), 16200 Belfort –El Harrach Algiers, Algeria; 3 National Institute of Agronomic Research–ASTER–Mirecourt (INRA), 662 AV Louis Buffet, 88500 Mirecourt, France

**Keywords:** Lactating Dairy Cow, Water Restriction, Blood Parameters, Total Dry Matter Intake, Mediterranean Climate

## Abstract

**Objective:**

This study was conducted to evaluate the effect of water restriction (WR) on physiological and blood parameters in lactating dairy cows reared under Mediterranean climate.

**Methods:**

The trial lasted 16 days preceded by two weeks of adaptation to the experimental condition in spring 2014 on 6 dairy cows in mid-lactation. These cows were allowed water *ad libitum* for 4 days (W100) (hydration period), then split into 2 groups, one group has received 25% and the other 50% of water compared to their mean water consumption during the hydration period; then rehydrated for 4 days. Feed intake and physiological parameters: respiratory rate (RR), heart rate (HR), and rectal temperature (RT) were recorded twice a day. Blood was collected once a day and analyzed for serum concentration of glucose (Glc), triglycerides (TG), cholesterol (Chol), urea (Ur), creatinine (Crea), and total protein (TP) by enzymatic colorimetric method and cortisol (Cort) by radioimmunoassay.

**Results:**

Total dry matter intake (TDMI) was affected by WR. A decrease in TDMI was observed in WR groups compared to W100 group (effect, group, period, day, group×day, period× day: p<0.001). Also, WR resulted in a significant increase in RR, HR, RT in WR groups than in W100 group (effect, group: p<0.001). In addition, an increase in the serum concentration of Glc, TG, Chol, Ur, Crea, TP, and Cort was noted in WR groups (effect, group, period, day: p<0.001).

**Conclusion:**

This study has shown the ability of cows raised in a Mediterranean climate to cope with different levels of WR and thus reach a new equilibrium. As result, elucidates the important role of water as a limiting factor for livestock in environments with low water availability.

## INTRODUCTION

Water is the most important nutrient for maintenance and productivity of dairy cows and contributes to all vital functions of the body (digestion, absorption, metabolism and transport of nutrient, elimination of wastes and excessive heat from the body). Its consumption can vary considerably depending on the type and size of the animal, the physical state, the activity level, dry matter intake (DMI), water quality, water temperature and ambient temperature [1].

Thus, any changes in its availability may lead to direct alteration in behavior of animals [2]. The latter, have developed different behavioral and physiological adaptation mechanisms which enable them to tolerate dehydration [3]. Indeed, these adaptation reactions represent a modification of ongoing physiological mechanisms in progress that allow an animal to respond to stress stimuli with minimum alteration in homeostasis [4], mainly in ruminants, especially in cattle, which are very sensitive to water scarcity than other domestic animals [5]. As a result, several studies have shown that water deprivation or restriction leads to a decrease in feed intake [6,7], metabolic rate during dehydration and endocrine balance [8]. On the other hand, it increases certain metabolites [9,10], hematocrit [11], respiratory rate (RR) [12], rectal temperature (RT) [13], and Cortisol [14].

Water as a natural resource, becomes more and more important, taking into account environmental issue, growing use and contrasting availability of the resource [15]. Its absence is a limiting factor for the development of livestock and its sustainability especially in countries with poor water resources and arid climate like Algeria. In this perspective, our main objective was to view how these cows can cope with a water restriction (WR) at two levels (W25, W50%). As a result, it impact on physiological, metabolic and hormonal parameters of lactating dairy cows.

## MATERIALS AND METHODS

### Animals and experimental procedures

An experiment was performed on six dairy cows in mid-lactation (144 to 150 postpartum) at experimental farm of Technical Institute Breeding (ITELV) of Baba Ali, located at 20 km south capital Algiers at 36° 65′ North latitude and 3° 06 East longitudes. The experiment was carried out under spring season (15 April to 16 May 2014) with mean daily temperature–humidity index (THI) value of 69.77±2.67 (without heat stress, excepted the last 8 days of experiment from 9 May to 16 May 2014) where the maximum temperature (Ta) exceeded 25°C, maximum humidity with 95% and THI>72%) revealing moderate heat stress [16]. This trial lasted 16 days, preceded by two weeks of adaptation to the experimental conditions. Before experiment, the cows were housed in semi covered free stall barn with the remaining herd. They were fed with dry fodder (3 boots of oat hay of 25 kg each, either 12.5 kg/cow/d; green fodder (30 kg of alfalfa/cow/d) and concentrate (6 kg/cow/d) with *ad libitum* access to drinking water.

**Figure 1:**
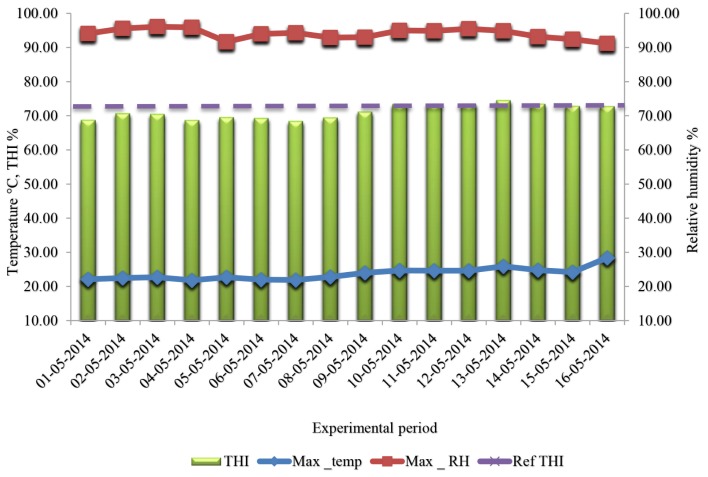
Average daily temperature, humidity and temperature–humidity index variation during experiment.

During the adaptation period (pre-experimental period), the cows were housed in covered tie stall barn with straw bedding and automatics waterers connected to individual water meters (*ad libitum* water). Feeding and milking were done twice a day (04:00 h; 16:00 h). During this period, no measures were performed on cows, only an adaptation to experimental conditions and feeding. The barn was equipped with a recorder thermo-hygrometer (Volt craft DL-120TH, Hirschau Bavière, Germany), connected to a portable PC programmed to record climatic parameters: ambient temperature (Ta) and relative humidity (RH), every hour during the whole experiment. The collected data were used to calculate the THI to characterize the climatic and environmental conditions which the animals were exposed to (Figure 1).

During experiment, cows had *ad libitum* access to water only during the first 4 days (hydration period: W100 group). In this period, food and water intake were recorded daily due to the presence of automatics waterers linked to the individual water meters. In restriction period (8 days), cows were assigned into two groups (matched by the food intake, milk yield, and water intake) that were subjected during 8 days to 25 and 50 restriction of drinking water relative to *ad libitum* intake (W100).

In restriction groups (W25; W50), cows received drinking water daily from 4:00 h at the same time when food was presented in the morning, until the allotted amount of drinking water was completely consumed; this was achieved by restricting water intake by 25 and 50 compared to the intake of the W100 group.

The restriction period was followed by a rehydration period of 4 days with *ad libitum* access to drinking water (W100).

### Measurement, sampling and laboratory analysis

Ta and RH were recorded twice per day. Maximum and minimum temperatures, and RH were recorded at 4:00 h and 16:00 h using a recorder thermo-hygrometer (Volt craft DL-120TH, Germany), daily THI values were also determined for the experimental period using the equation as described by Kibler [17].

THI (%)=1.8×Ta-(1-RH)×(Ta-14.3)+32

Where, Ta, average ambient temperature in °C and RH, average relative humidity in (%).

To determine the DMI, the amounts of the feed offered and refused were recorded daily throughout the experiment. Refused feed was removed and weighed daily just prior to the morning feeding. All cows consumed all of the concentrate; therefore, weigh-back consisted of only oat hay and alfalfa. The samples of feed and refusal were taken daily and one fraction was used for DM determination by drying at 105°C in a forced air oven for 24 h at central laboratory of experimental farm of Baba Ali (ITELV).

Physiological parameters were recorded twice per day (4:00 am; 16:00 pm): RR, heart rate (HR), and RT of cows were measured just prior to each milking. RT was measured by inserting a veterinary digital thermometer at about 60 mm into the rectum for 60 s. HR was determined using a stethoscope for one minute. RR was measured by counting the flank movements of the individual cows for a 1 min period of uninterrupted breathing.

Blood samples were taken each morning (4:00 h) from the jugular vein using heparinized vacutainer tubes before the cows had been milked and fed. The samples were kept in a cooler at 4°C for a few hours until their shipment to the Higher National Veterinary School of Algiers (ENSV) where they will be immediately centrifuged (3,500 rpm for 15 min) to recover the plasma. Plasma samples were then frozen at –20°C for hormone analysis. Blood plasma were analyzed for free Cortisol (Cort) using a radioimmunoassay kit (Immunotech, ref.1363). Cort concentration was estimated by radioimmunoassay using the diagnostic 125 I kit supplied by Immunotech-Radiova 1, Prage, Czerch Republic) suitable for the quantitative determination of Cortisol levels in serum, plasma or urine. These analyzes were performed at the endocrinology laboratory of Houari Boumedienne, University of Science and Technology, Babe-El Zouar (USTHB), Algiers.

Metabolic parameters (glucose [Glc], cholesterol [Chol], triglyceride [TG], urea [Ur], creatinine [Crea], total protein [TP]), were determined by an automated Biochemistry Analyzer (Mindray BA-88, Nanshan, Shenzhen, China) using commercial kit (SPINREACT, S.A.U, Ctra Santa Coloma, Spain), performed in the central laboratory of technical institute of breeding (ITELV).

### Statistical analysis

To determine the effect of group (water restriction W25, W50), period (hydration, restriction, and rehydration), day of observation and their interaction (group×day; period×day), we used a mixed model (MIXED PROC) of SAS software Version 9.4 (Institute Inc, Cary, NC, USA) where repeated measurements were performed per day. according to the following model:

Y=μ+G+P+CW+D+G×D+P×D+e

Where: Y = Dependent variable; μ = mean effect; G = effect of group; P = effect of period; D = effect of day; CW = effect of cow; G×D = interaction of group×day; P×D = interaction of period×day; e = the residual effect. Repeated day/sub = cow ×period, type = VC. Variance and covariance assumption structures including AR (auto regressive unstructured), and compound symmetry were tested. The data were performed as mean±standard error and the difference were considered significant at p<0.05.

## RESULTS

### Water intake

During the experiment, the WR caused a significant decrease in water intake (Table 1) on the 8 days of restriction (day 5 to 12 of the experiment). In fact, the WR group (W25, W50) have consumed respectively 42.66 L and 28.44 L compared to the mean water intake in the *ad libitum* W100 group (first 4 days) (58.66 L) (effect, group, period, day, group×day, period×day: p<0.001, Table 1). While during the last 4 days of the experiment, the mean water intake of the WR groups increased to 53.79 L, without reaching the hydration period level (W100)(effect, group, period, day, group×day, period×day: p<0.001, Table 1).

### Feed intake

Feed intake was affected also by WR (Table 1). Indeed, TDMI was decreased in the WR groups especially in W50 group (21.00±0.20) compared to those of W25 and W100 (effect, group×day, period×day: p<0.001, Table 1). Then, the TDMI of W50 group was recovered 24 hours after rehydration period (W100) (27.31±0.40) and has even exceeded that of hydration period (25.00±0.35) (effect, day: p<0.001, Table 1). The lowest TDMI observed in WR groups (W25, W50) resulted, from 08 days, in a progressive and significant (p<0.001) reduced intake of oat hay (6.41±0.35 kg W25; 9.58±0.35 kg W50) and alfalfa between the W50 (9.21±0.15 kg/d) and W100 group (10.40±0.13 kg/d). Slight effect on concentrate intake due to WR was found between WR groups (5.35±0.09 kg) and W100 group (5.01±0.09 kg) (effect, group: p<0.005, Table 1).

### Rectal temperature

Our results showed that WR has not affected RT (Table 2). Indeed, an increase in RTpm was observed in W50 group (38.91°C±0.06°C) than in that of W25 (38.32°C±0.04°C) (effect, group: p<0.001, Table 2). The increase in RTpm persisted during the rehydration period (38.75°C±0.07°C) and even exceeded that of hydration period (38.65°C±0.07°C), unlike at the RTam. But this elevation remains within the normal RT range required for dairy cows (38°C to 39°C).

### Respiratory rate

The RR was influenced by the WR. Indeed, an increase in RR was observed in WR groups than in *ad libitum* W100 group (p<0.001). This increase was more pronounced on afternoon RR (RRpm) particularly in WR groups (48.40±0.78 in W25; 50.83±1.17 in W50) (effect, group, period, day: p<0.01, Table 2) than in morning RR (RRam) (33.40±0.95 rate/min in W25; 40.80±0.95 rate/min in W50) (effect, group<0.001, Table 2). Then, the RR of WR groups decreased significantly whether during the rehydration (37.27±1.08 rate/min) and hydration period (36.76±0.97 rate/min) (effect, group: p<0.001, Table 2).

### Heart rate

The WR has influenced the HR (Table 2). Significant difference was observed in the morning and afternoon HR between WR groups and the *ad libitum* W100 group. Indeed, an increase in HR am was very pronounced in the W50 group (76.33±1.07 beats/min) than in W25 group (69.29±0.71 beats/min). This increase in HR has persisted during the rehydration period (77.82±1.09 beats/min) and has even exceeded the hydration period (W100) (71.30±0.98 beats/min) (effect, group, period, day, cow, group×day, period×day: p<0.001, Table 2). In contrast, HR pm has again increased in W25 group (74.20±1.70 beat/min) and W50 group (81.50±1.70 beats/min) compared to rehydration period (78.87±1.12 beats/min) that exceeded the hydration period (73.56±0.97 beats/min) (effect, group, period, day, group×day: p<0.001, Table 2). These frequencies did not exceed the values reference required for dairy cow (60 to 80 beats/min).

### Blood parameters

The WR significantly influenced the serum concentration of Glc, Chol, TG, TP, Ur, Crea, and Cort (Table 3).

Thus, serum Glc concentrations were significantly higher in WR groups (1.20±0.39 g/L in W25; 1.38±0.39 g/L in W50) compared to the *ad libitum* W100 group: rehydration (0.60± 0.04 g/L) and hydration (0.52±0.03 g/L) (effect, group, period, day, cow, group×day, period×day: p<0.001, Table 3).

Serum Chol concentrations exhibited significantly increased (p<0.001) values in WR groups (2.40±0.06 g/L in W25; 2.50 ±0.06 g/L in W50) compared to the *ad libitum* W100 group: rehydration (1.21±0.06 g/L) and hydration (1.40±0.05 g/L) (effect, group, period, day, cow, group×day, period×day: p< 0.001, Table 3).

Similarly, elevated plasma TG concentrations were recorded in WR groups (2.30±0.09 g/L in W25) and (2.40±0.09 g/L in W50) relative to the *ad libitum* W100 group: rehydration (0.95 ±0.09 g/L) and hydration (0.80±0.08 g/L) (effect, group, period, day, cow: p<0.001, Table 3).

Plasma concentrations of Crea have exhibited significantly increased (p<0.001) values due to the WR effect. This effect was very pronounced in WR groups (18.34±0.75 g/L in W25 and 19.3±0.75 g/L in W50) than in the *ad libitum* W100 group, whether during the rehydration (7.57±0.75 mmol/L) or the hydration period (6.60±0.67 mmol) (effect, group, period, day, cow, group×day, period×day: p<0.001, Table 3).

The Ur content markedly increased (p <0.001) in the WR groups (1.23±0.07g/L in W25 and 1.34±0.07 g/L inW50) compared to the rehydration and hydration (W100 group) which recorded the same value (0.25±0.06 g/L) (effect, group, period, day, cow, group×day, period×day: p<0.001, Table 3).

Indeed, a very significant increase in the plasma TP content was recorded in the WR groups (89.70±1.13 g/L in W25 and 90.91±1.13 g/L in W50) compared to the rehydration (67.90±1.09 g/L) and hydration period (65.68±0.97 g/L) (effect, group, period, day, cow: p<0.001, Table 3).

Plasma Cort concentrations were also affected by WR (p < 0.001). In fact, an increase in plasma Cort concentrations were observed in WR groups: W50 (37.70±1.12 nmol/L) and W25 (24.30±1.12 nmol/L) compared to the rehydration period (12.38±1.46 nmol/L) (effect, group, period, day, cow, group× day: p<0.001, Table 3).

## DISCUSSION

Several studies carried out in various animal species (dairy cows, sheep, goats) have shown their ability to tolerate different regimes of WR through various mechanisms [6,18]. Indeed, a significant reduction in DMI under WR was linked, on one hand, to the type of feed that animals received [19], and on the other hand, to the decrease in the quantity of drinking water intake. Our results were similar to those of Williams et al [20] who’s showed that cows prefer to have access to water during feeding and any WR affects negatively the amount of feed intake. Similarly, Kramer et al [21] and Kume et al [22] have shown that WR was positively correlated with feed intake. This fed reduction was explained by the fact that it be partially offset by reduced intestinal peristalsis, which leads to an increased time of exposure of feed to the intestinal micro flora with beneficial effects on digestibility and feed utilization [23].

The increase in RT in our study was a modest response under the combined effect of moderate heat stress and dehydration (WR), but it remains within standards range of RT for dairy cows (38°C to 39°C). Also, this increase in RT saves on water losses. Our results were an agreement with Alamer [7] in Aardi goats and Ghanem [11] in Lacaune ewes that have shown no change in RT under WR.

The grow in RR, especially in afternoon was directly related to the rise in ambient temperature (>25°C) and RH intensified by WR particularly in the last 4 days of restriction period response to a moderate heat stress (THI>72%). Our results were similar to those of Du Preez [24]. Ghassemi Nejad and Sung [4], have also shown that physiological changes including rise to respiratory and HR were indicators of heat stress.

Similarly, the difference in HR was related to the increase in ambient temperature (> 25°C) associated with a high rate of humidity (94%). As a result, THI>72% revealing moderate heat stress [25]. Our results confirm those of Stockman [26], who has showed an increase in HR in response to a warm environment.

WR has affected significantly the serum Glc, TG, Chol, Ur, Crea, TP, and Cort concentrations. This elevation was very pronounced in the WR groups (WR25, WR50) compared to those watered at will (W100). Our results corroborate those of Kaliber et al [27] in goats but opposed to those of Burgos et al [6] in dairy cows, which showed a decrease in Glc concentrations.

Regarding to serum concentrations of metabolites, our results opposed respectively to those of Burgos et al [6] in dairy cows and Ghanem [11] in Lacaune ewes, which showed no effect of WR on plasma TG concentration and Chol (p< 0.05).

A pronounced increase in serum Crea concentrations were observed in our study. Our results were similar to those of Ghanem [11] in Lacaune ewes and Abd Elatif et al [28] in Barki sheep.

For serum Ur concentrations, our results coincide with those of Burgos et al [6] in dairy cow and Ghanem [11] in the Lacaune ewes, which also found high Ur levels in the WR groups compared to the hydrated group. This increase in serum Ur concentrations were attributed to the restricted water condition that produced a dehydration state with hematological concentration of the metabolites.

Likewise, a marked increase in serum TP concentrations was observed in our trial. So, our results were in contrast to those of Burgos et al [6], which found no effect of WR in serum concentrations of TP content. On the other hand. They coincide with those of Ghanem [11], which found significant increase in serum TP in WR groups (82.0 g/L) than in rehydration group (72.7 g/L). Also, the same findings was observed by Mengistu et al [29] in Ethiopian-Somali goats with water for four days.

A significant increase in serum Cort concentrations was noted in the WR restricted groups compared to those watered at will. This was a normal response to prolonged WR (8 days). Since, Cort plays an important role in maintaining fluid balance and plasma electrolytes [30]. This, joins the results of several authors [14,10]. This rise can also be explained by the increase in ambient temperature (>25°C) associated with a high humidity (95%) and THI>72% revealing a moderate stress associated with WR. But, our results differ from those of Burgos et al [6] in dairy cows, which showed a decrease in plasma Cort concentration during a WR of 8 days.

## Figures and Tables

**Table 1:** Mean water and feed intakes of hydration, rehydration period and water-restriction dairy cow

Item	Groups				Effect					

	Hydration	Restriction period		Rehydration						
			
	W100	W25	W50	W100	G	P	D	CW	G×D	P×D
WI (L/d)	56.88±1.04^a^	42.66±0.79^b^	28.44±0.79^c^	53.79±1.24^d^	***	***	***	***	**	***
DMI (kg/d)										
HDMI	11.36±0.36^a^	9.58±0.35^b^	6.41±0.35^c^	10.27±0.41^a^	***	***	***	***	***	***
ADMI	8.30±0.11^a^	9.50±0.19^b^	9.21±0.15^b^	10.40±0.13^c^	ns	***	***	ns	ns	***
CDMI	5.16±0.08^a^	5.35±0.09^b^	5.35±0.09^b^	5.01±0.09^c^	*	**	***	***	ns	ns
TDMI	25.00±0.35^a^	24.41±0.32^a^	21.00±0.20^b^	27.31±0.40b,a	***	***	***	***	***	***

G, group effect; P, period effect; D, day effect; CW, cow; G×D, interaction of water restriction and day; P×D, interaction of period and day; WI, water intake; DMI, dry matter intake; HDMI, dry matter intake of oat hay; ADMI, dry matter intake of alfalfa; CDMI, dry matter intake of concentrated; TDMI, total dry matter intake.

abcd Values within a row with different superscripts differ significantly.

* p<0.05;

** p<0.01;

*** p<0.001;

ns, not significant.

**Table 2:** Effect of water restriction on physiological parameters of lactating dairy cows reared under in a Mediterranean climate

Parameters	Groups				Effect					

	Hydration	Restriction period		Rehydration						
			
	W100	W25	W50	W100	G	P	D	CW	G×D	P×D
RRam (rate/min)	36.76±0.97^a^	33.40±0.95^b^	40.80±0.95^c^	37.27±1.08^a^	***	ns	ns	***	ns	ns
RRpm (rate/min)	46.67±1.02^a^	48.40±0.78^a^	50.83±1.17^a^	41.73±1.14^b^	***	***	***	**	ns	ns
HRam (beats/min)	71.30±0.98^a^	69.20±0.71^a^	76.33±1.07^b^	77.83±1.09^b^	***	***	***	***	***	***
HRpm (beats/min)	73.56±0.97^a^	74.20±1.07^a^	81.50±1.07^b^	78.87±1.12^c^	***	***	***	***	ns	***
RTam (°C)	38.28±0.05^a^	38.00±0.051^b^	38.50±0.05^c^	38.27±0.06^a^	***	ns	ns	***	ns	ns
RTpm (°C)	38.65±0.07^a^	38.32±0.04^b^	38.91±0.06^c^	38.75±0.07^a^	***	ns	ns	***	ns	ns

W100, water *ad libitum*; W25, water restriction at 25%; W50, water restriction at 50%; G, water restriction effect; P, period effect; D, day effect; CW, cow; G×D, interaction of water restriction and day; P×D, interaction of period and day; RRam, morning respiratory rate; RRpm, evening respiratory rate; HRam, morning heart rate; HRpm, evening heart rate; RTam, morning rectal temperature; RTpm, evening rectal temperature.

abc Values within a row with different superscripts differ significantly.

** p<0.01,

*** p<0.001;

ns, not significant.

**Table 3:** Blood parameters of hydration and rehydration period, and water restricted dairy cows

Items	Groups				Effect					

	Hydration	Restriction period		Rehydration						
			
	W100	W25	W50	W100	P	G	D	CW	G×D	P×D
Clc (g/L)	0.52±0.03^a^	1.20±0.39^b^	1.38±0.39^c^	0.60±0.04^d^	***	***	***	ns	***	***
TG (g/L)	0.84±0.08^a^	2.30±0.09^b^	2.40±0.09^b^	0.95±0.09^c^	***	***	***	***	***	ns
Chol (g/L)	1.40±0.05^a^	2.40±0.06^b^	2.50±0.06^b^	1.21±0.04^c^	***	***	***	***	ns	***
TP (g/L)	65.68±0.97^a^	89.70±1.13^b^	90.91±1.13^b^	67.90±1.09^a^	***	***	***	ns	***	ns
Ur (mmol/L)	0.25±0.06^a^	1.23±0.07^b^	1.34±0.07^c^	0.25±0.06^a^	***	***	***	***	ns	***
Crea (mmol/L)	6.60±0.67^a^	18.34±0.75^b^	19.30±0.75^b^	7.57±0.75^a^	***	***	***	***	***	***
Cort (nmol/L)	15.57±1.31^a^	24.30±1.12^b^	37.70±1.12^b^	12.38±1.46^d^	***	***	***	**	ns	***

W100, hydration period; W25, water restriction at 25%; W50, water restriction at 50%; W100, rehydration period; P, period effect; G, water restriction effect; D, day effect; CW, cow; G×D; interaction of water restriction and day; P×D, interaction of period and day; Glc, glucose; TG, triglyceride; Chol, cholesterol; TP, Total protein; Ur, Urea; Crea, creatinine; Cort, cortisil.

abcd Values within a row with different superscripts differ significantly.

** p<0.01,

*** p<0.001;

ns, not significant.
